# The Hekinan Children’s Study: Design and Profile of Participants at Baseline

**DOI:** 10.2188/jea.JE20180005

**Published:** 2019-07-05

**Authors:** Chisato Nagata, Keiko Wada, Yukari Sahashi, Takashi Tamura, Kie Konishi, Yuko Goto, Michiyo Yamakawa, Sachi Koda, Fumi Mizuta, Takahiro Uji, Kozue Nakamura, Michiko Tsuji, Hideshi Nagai, Naoko Itakura, Kou Harada, Osamu Takahara, Hiromichi Yamanaka

**Affiliations:** 1 Department of Epidemiology and Preventive Medicine, Gifu University Graduate School of Medicine, Gifu, Japan; 2 Department of Preventive Medicine, Nagoya University Graduate School of Medicine, Nagoya, Japan; 3 Department of Food and Nutrition, Gifu City Women’s College, Gifu, Japan; 4 Department of Food Science and Nutrition, Nagoya Women’s University, Nagoya, Japan; 5 Hekinan Medical Association, Hekinan, Aichi, Japan

**Keywords:** profile, school children, cohort studies, Japan

## Abstract

**Background:**

The early life environment is now recognized as a key factor contributing to susceptibility to certain diseases in later life.

**Methods:**

We initiated a cohort study among school children in 2011 to primarily investigate the associations between lifestyle and environmental factors and some surrogate markers of chronic diseases, such as cardiometabolic risk factors (ie, obesity, high blood pressure, high blood glucose, insulin, or lipids) and cancer risk factors (ie, height and age at menarche). A baseline questionnaire asked for information, including demographic variables, medical history and use of medication, dietary habits, physical activity, sleep habits, and behavioral and emotional problems of children. Follow-up surveys are planned for the fourth grade of elementary school and the first grade of junior high school. At these follow-up surveys, fasting blood samples will be obtained to measure cardiometabolic markers. We also checked the validity of a food frequency questionnaire, which was originally created for 6-year-olds but was modified for use in older children.

**Results:**

A total of 3,141 first-year students at elementary schools in Hekinan City, Aichi Prefecture, participated in the study. The response rate was 87.4%. The means of age and body mass index were 6.99 (standard deviation, 0.28) years and 15.3 (standard deviation, 1.7) kg/m^2^, respectively, in the 3,067 Japanese children (1,639 boys and 1,428 girls).

**Conclusions:**

This cohort will reveal determinants of cardiometabolic risk factors and cancer risk factors during childhood.

## INTRODUCTION

Chronic lifestyle-related diseases, such as cardiovascular diseases and cancer, are major causes of death among Japanese. The early life environment is now recognized as a key factor contributing to susceptibility to these diseases later in life.^1^^,^^2^ This hypothesis has proven difficult to test, as disease outcomes occur decades later. However, the use of proxy outcomes that lie on the causal pathway linking an exposure to the risk of the diseases could potentially identify important predictors or determinants of later health and disease.^3^ Several epidemiologic studies have shown that the atherosclerotic process begins in childhood, and individual cardiovascular risk factors, such as obesity, blood pressure, blood lipid, and glucose levels, tend to persist over time from childhood to adulthood.^4^^–^^8^ Established risk factors for certain cancers emerge in the early stages of life: for example, height for colorectal,^9^ breast,^9^ and prostate^10^ cancers and age at menarche for breast cancer.^11^ Child cohort studies can have advantages because they can capture such surrogate endpoints in early life. To date, epidemiologic research has focused primarily on adult populations. Several child cohort studies are now ongoing in Japan.^12^^–^^19^ However, the accumulating evidence for the role of early life exposures is still insufficient. In particular, dietary intake has not been commonly assessed in previous cohort studies, probably because there are few questionnaires validated for use among Japanese children.^20^^,^^21^ We launched the Hekinan Children’s Health Study in 2011 to primarily investigate the associations between lifestyle and environmental factors and surrogate markers of chronic diseases, such as cardiometabolic risk factors and cancer risk factors, among school children in Hekinan City, Aichi Prefecture. Our study pays special attention to the potential role of diet as a determinant of these surrogate endpoints. As secondary endpoints, we include allergies, pubertal development, myopia, back and neck pain, and other health conditions. As lifestyles, including eating behaviors, are thought to track across childhood and into adulthood,^22^^,^^23^ we also assessed changes in environmental and lifestyle behaviors, as well as surrogate markers, during follow-up. Here, we describe the study’s design and the profile of participants at the study’s baseline. An additional aim of the present study is to validate a food frequency questionnaire (FFQ) for use in follow-up on participants’ diet.

## METHODS

### Study participants

We recruited a cohort that consists of first-year students at elementary schools in Hekinan City, Aichi Prefecture. Hekinan City is located in central Aichi Prefecture and is within 40 km of Nagoya City. As of March 2010, the population was 72,018, and 4.3% of workers were employed in primary industries, 47.1% in secondary industries, and 45.1% in tertiary industries. Hekinan City has the relatively warm climate (the overall mean temperature was 16.6°C in 2010). The survey covered all schools (*n* = 7) in the city. Information sheets and consent forms were distributed to 3,594 parents via the schools, where they were informed and invited to let their children participate in the present study. Informed consent was obtained from each parent. The study was approved by the ethics board of the Gifu University Graduate School of Medicine.

### Baseline survey

The baseline survey was conducted from September 2011 through October 2015. A total of 3,141 students participated in the study. The participation rate was 87.4%. The annual response rates for 2011 through 2015 were 87.4, 83.2%, 89.6%, 89.7%, and 87.0%, respectively. The parents completed a questionnaire asking birth size, height, and weight at the 4-month, 1.5-year, and 3-year health check-ups provided by the city; number of siblings; health status, including major diseases and the frequency of common colds or fever; use of medication or supplements; use of sunscreen; dietary intake and diet behavior; physical activity and sedentary behavior; sleep habits; use of cellular phones; and behavioral and emotional problems of children. For the common colds, symptoms like clogged or running nose, a sore throat, and coughing were given in the questionnaire.^24^ Sociodemographic characteristics and medical histories of parents and the smoking habits of parents and other family members were also asked. For parents who were non-Japanese (*n* = 74), a different version of the questionnaire that excluded several questions was utilized. Data on children’s heights and weights were obtained through health check-ups conducted at the schools.

Dietary intake was assessed using a FFQ validated previously for 6-year-old children. The FFQ asked parents how often, on average, their children consumed each of the 162 food items listed and what the usual serving size of each item was during the 6 months prior to the study. The validity of the FFQ was assessed by comparison with two 3-day diet records; the details of the validation study have been described elsewhere.^25^ The dietary behaviors of respondent’s children—such as the frequency and timing of breakfast, lunch, dinner, and snacking; frequency of fast food consumption; speed of eating; and who dines with their children—were also asked.

Referring to an outdoor playtime checklist by Burdettes et al,^26^ physical activity was assessed by asking how much time before noon, from noon until 6 pm, and after 6 pm the child spent playing in the yard or street around the house and in the park and playground or outdoor recreation area. These questions were asked for both weekday and weekend activities. Playtime categories (0, 1–15, 16–30, 31–60, or ≥61 minutes) were provided as response options for each question. They were scored as 0 for 0 minutes to 4 for ≥60 minutes, and the sum of these scores was regarded as the physical activity score (the maximum score was 40). The details, including reproducibility, have been described elsewhere.^27^ Information regarding the child’s participation in organized sports, games, and other activities supervised by adults was also sought. Parents were asked to identify each organized physical activity in which the child was involved, the average number of times per week the child participated, and the average duration of participation. These questions were adapted from the questionnaire by Booth et al.^28^ Time spent in moderate-to-vigorous physical activity (METs ≥3.0) was calculated for each child. Each child’s behavioral and emotional problems were assessed using a Strength and Difficulties Questionnaire.^29^

Each question was checked for implausible and missing values, and correct information was sought again from parents whenever possible. Parents were also asked to provide their child’s first morning void of urine. Urine was collected at home and brought to the school on the same day. Urine samples were aliquoted at the same day and stored at −80°C until assayed. Biomarkers of passive smoking, vitamins, flavonoids, trace elements, hormones, and antioxidants will be measured using urine samples.

### Follow-up surveys

Follow-up surveys are planned for the fourth grade of elementary school and the first grade of junior high school. The second and third surveys will include anthropometric measurements, blood pressure measurements, and fasting blood analysis. Fasting blood samples will be obtained at heath check-ups at the schools, aliquoted on the same day, and stored at −80°C until assayed. Follow-up questionnaires will ask questions similar to those on the baseline questionnaire; however, some questions will be modified, considering the age of the children. For example, questions assessing physical activity will be modified as demonstrated by Booth et al.^27^ The questions have two main components: participation in organized sports, games, and other activities; and participation in non-organized physical activities. Similar to the baseline questionnaire, organized physical activities are defined as those that usually involve competition, are supervised by adults, and involve training/coaching. Baseball, swimming, or football team sports and dance classes are given as examples. Non-organized activities are defined as those that do not involve regular training or competition, do not have a coach, and do not usually involve adult supervision. Walking, cycling, jumping rope, and casual ball games are examples. Parents are asked to report every activity in which their child participated and the frequency and average duration of each activity. The onset of menarche is asked in the follow-up questionnaire. The questionnaire also includes questions concerning secondary endpoints, such as puberty, back or neck pain, and obstructive sleep apnea. Puberty status is measured using scales developed by Chan et al.^30^ We plan to administer the questionnaire to the children themselves. Some questions overlap with those for parents. Depressive status and some questions regarding sleep habits are only asked of children. Depressive status is measured using the Japanese version of the Birleson Depression Self-Rating Scale for Children.^31^ Data on height and weight at annual health check-ups and results from physical fitness tests conducted at the baseline and at the time of follow-up surveys will be obtained from schools.

### Validation of FFQ

As the FFQ used in the baseline survey was created for use in children 6 years or under, we created a modified version of the FFQ by changing the portion size of the food items and adding new food items that were more likely to be consumed in older children, such as coffee and coffee products. We assessed the validity of this modified FFQ in a sample of 268 children aged 10–11 (139 boys and 135 girls; mean body mass index, 17.1 kg/m^2^ [standard deviation {SD}, 2.1]) in the seven schools in 2009. Parents completed a 3-day diet record, including two successive weekdays and one weekend, in April 2009. The lunch menus corresponding to the weekdays were obtained from the schools, and parents were asked how much food their children left on their plates. The FFQ was administered in May–June 2009. The correlation coefficients between the diet record and the FFQ for intakes of nutrients and food groups were calculated. Total energy was adjusted using the residual method.

## RESULTS

Baseline characteristics of the 3,067 Japanese study participants are summarized in Table 1. Out of the 3,067 participants, 3,055 responded to the baseline FFQ. The means of the daily intake of nutrients and food groups among the participants, after excluding those whose energy intake was less than 500 kcal (*n* = 0) or greater than 3,500 kcal (*n* = 24), are shown in Table 2. These cut-off values were determined referring to those in previous studies.^20^^,^^32^ Characteristics and the daily intake by sex are presented in eTable 1, eTable 2, eTable 3, and eTable 4.

**Table 1.** Characteristics of 3,067 study participants at baseline

	Number of valid response	Mean (SD)	Number (%)
Year	3,067		
2011			666 (21.7)
2012			581 (18.9)
2013			645 (21.0)
2014			606 (19.8)
2015			569 (18.6)
Age, years	3,067	6.99 (0.28)	
Sex	3,067		
Girls			1,428 (46.6)
Boys			1,639 (53.4)
Height, cm	3,049	118.0 (4.9)	
Weight, kg	3,049	21.4 (3.5)	
BMI, kg/m^2^	3,049	15.3 (1.7)	
Height at birth, cm	2,951	49.1 (2.5)	
Weight at birth, g	2,987	2,982 (442)	
Height at 4 months, cm	2,858	62.3 (2.9)	
Weight at 4 months, kg	2,861	6.7 (0.9)	
Height at 1.5 years, cm	2,806	79.7 (3.1)	
Weight at 1.5 years, kg	2,817	10.3 (1.1)	
Height at 3 years, cm	2,759	92.8 (3.4)	
Weight at 3 years, kg	2,764	13.5 (1.6)	
Number of sibling	3,056	1.4 (0.8)	
Breast feeding^a^	3,013		2,893 (96.0)
Users of cellar phone	3,061		222 (7.3)
Users of dietary supplements	3,016		250 (8.3)
Nonusers of sunscreen	3,052		1,801 (59.0)
Medical history			
Heart or kidney disease	3,058		117 (3.8)
Digestive diseases	3,056		20 (0.7)
Asthma	2,949		350 (11.9)
Hospitalization in the past year	3,045		128 (4.2)
Number of visits to clinics in the past year	2,798	7.4 (9.4)	
Number of fever episodes in the past year	3,022	2.4 (2.2)	
Number of common cold events/influenza infection in the past year	3,011	3.7 (3.9)	
Diet			
Breakfast			
Not every day	3,053		202 (6.6)
Eat alone	3,042		163 (5.4)
Timing at weekdays	3,017	6:48 a.m. (0:17)	
Timing at weekends	2,963	7:50 a.m. (0:41)	
Dinner			
Not every day	3,054		10 (0.3)
Eat alone	3,044		2 (0.07)
Timing at weekdays	3,016	6:34 p.m. (0:37)	
Timing at weekends	2,976	6:35 p.m. (0:36)	
Fast foods, ≥once a week	3,058		170 (5.6)
Sleep			
Wake-up time at weekdays	3,059	6:31 a.m. (0:18)	
Wake-up time at weekends	3,053	7:17 a.m. (0:44)	
Bed-time at weekdays	3,061	9:11 p.m. (0:35)	
Bed-time at weekends	3,057	9:28 p.m. (0:40)	
Duration of sleep at weekdays, hours	3,057	9.3 (0.6)	
Duration of sleep at weekends, hours	3,047	9.8 (0.7)	
Physical activity			
Moderate-vigorous, h/wk	3,047	1.65 (2.52)	
Playtime score	3,005	10.9 (6.1)	
Strength and Difficulties Questionnaire score	3,059	10.6 (5.2)	
Fathers			
Age, years	2,882	38.5 (5.4)	
Height, cm	2,850	170.8 (5.8)	
BMI, kg/m^2^	2,700	23.4 (3.3)	
Years of education ≥16 years	2,824		772 (27.3)
Current smokers	2,897		1,324 (45.7)
Ex-smokers	2,897		722 (24.9)
Mothers			
Age, years	3,022	36.4 (4.7)	
Height, cm	3,006	157.5 (5.3)	
Weight, kg	2,855	52.3 (8.1)	
BMI, kg/m^2^	2,853	21.1 (3.1)	
Years of education ≥16 years	2,976		503 (16.9)
Current smokers	2,979		382 (12.8)
Ex-smokers	2,979		429 (14.4)

BMI, body mass index; SD, standard deviation.

^a^Including mixed feeding.

**Table 2.** Daily nutrient and food intakes among 3,031 children

	Mean (SD)
Energy, kcal	1,633 (435)
Protein, g	61.3 (17.4)
Total fat, g	54.8 (16.8)
Saturated fat, g	19.4 (6.1)
Monounsatulated fat, g	18.6 (6.0)
Polyunsatulated fat, g	10.0 (3.3)
Cholesterol, mg	280 (99)
Carbohydrate, g	221 (59)
Calcium, mg	689 (283)
Magnesium, mg	221 (68)
Phosphorus, mg	1,047 (311)
Iron, mg	6.4 (2.1)
Zinc, mg	7.6 (2.1)
Sodium, mg	3,029 (1012)
Potassium, mg	2,396 (760)
Vitamin A,^a^ µg	642 (257)
Retinol, µg	312 (189)
α-carotene, µg	739 (323)
β-carotene, µg	3,379 (1412)
Cryptoxantin, µg	351 (485)
Carotene,^b^ µg	3,935 (1623)
Vitamin B1, mg	0.91 (0.27)
Vitamin B2, mg	1.26 (0.34)
Vitamin B6, mg	1.09 (0.34)
Vitamin B12, µg	5.57 (2.51)
Folate, µg	279 (100)
Niacin, mg	12.7 (4.0)
Vitamin C, mg	93 (43)
Vitamin D, µg	6.2 (2.7)
α-tocopherol, mg	6.1 (2.1)
Dietary fiber, g	11.3 (3.9)
Salt, g	7.7 (2.6)
Cereals/potatoes/starches, g	295 (89)
Soy products, g	55.2 (42.7)
Fishes and shellfishes, g	44.5 (21.1)
Meats, g	64.4 (27.1)
Eggs, g	28.7 (15.3)
Milk and dairy products, g	341 (184)
Green and yellow vegetables, g	61.3 (30.7)
Other vegetables, g	186 (86)
Algae, g	3.8 (3.3)
Fruits, g	99 (98)
Confectioneries, g	72 (55)

SD, standard deviation.

^a^Retinol equivalents.

^b^β carotene equivalents.

The results from the validation study of the FFQ for follow-up are shown in Table 3. For most nutrients and food groups, the mean intakes estimated from the FFQ were higher than those derived from the diet records. The Spearman correlation coefficients between the diet records and the FFQ ranged from 0.11 for cryptoxanthin to 0.51 for dairy foods. Adjustment for energy intake somewhat raised the correlations. Adjustment for sex and school did not alter the results (data not shown).

**Table 3.** Daily nutrient intakes assessed with two 3-day diet records and FFQ and their correlations

	3-day diet records	FFQ	Spearman correlation coefficients	Spearman correlation coefficients^c^
	
	Mean (SD)	Mean (SD)		
Energy, kcal	1,934 (282)	2,207 (523)	0.28^**^	
Protein, g	67.3 (11.2)	83.6 (22.0)	0.32^**^	0.27^**^
Total fat, g	60.9 (14.2)	72.1 (21.3)	0.28^**^	0.38^**^
Saturated fat, g	20.7 (5.6)	24.4 (7.3)	0.26^**^	0.33^**^
Monounsatulated fat, g	21.1 (5.5)	25.0 (7.9)	0.27^**^	0.36^**^
Polyunsatulated fat, g	12.0 (3.7)	13.7 (4.2)	0.20^**^	0.29^**^
Cholesterol, mg	316 (109)	392 (135)	0.38^**^	0.41^**^
Carbohydrate, g	272 (42)	301 (71)	0.25^**^	0.37^**^
Calcium, mg	633 (178)	825 (309)	0.35^**^	0.45^**^
Magnesium, mg	223 (41)	297 (87)	0.34^**^	0.45^**^
Phosphorus, mg	1,061 (186)	1,361 (365)	0.33^**^	0.37^**^
Iron, mg	6.7 (1.5)	8.9 (2.7)	0.25^**^	0.27^**^
Zinc, mg	8.5 (1.6)	10.3 (2.6)	0.30^**^	0.25^**^
Sodium, mg	3,099 (531)	4,137 (1239)	0.28^**^	0.30^**^
Potassium, mg	2,235 (460)	3,067 (913)	0.34^**^	0.45^**^
Vitamin A,^a^ µg	566 (648)	849 (325)	0.27^**^	0.37^**^
Retinol, µg	286 (615)	440 (244)	0.28^**^	0.32^**^
α-carotene, µg	686 (534)	907 (359)	0.19^**^	0.28^**^
β-carotene, µg	2,916 (1473)	4,130 (1596)	0.30^**^	0.36^**^
Cryptoxantin, µg	77.7 (166.6)	566 (730)	0.11	0.07
Carotene,^b^ µg	3239 (1485)	4875 (1910)	0.28^**^	0.28^**^
Vitamin B1, mg	0.88 (0.21)	1.23 (0.36)	0.14^*^	0.15^*^
Vitamin B2, mg	1.26 (0.34)	1.65 (0.50)	0.30^**^	0.35^**^
Vitamin B6, mg	1.09 (0.26)	1.49 (0.44)	0.25^**^	0.35^**^
Vitamin B12, µg	6.32 (4.91)	8.05 (3.26)	0.21^**^	0.19^**^
Folate, µg	259 (79)	358 (123)	0.34^**^	0.40^**^
Niacin, mg	13.3 (3.2)	18.0 (5.5)	0.28^**^	0.27^**^
Vitamin C, mg	95 (82)	115 (51)	0.30^**^	0.41^**^
Vitamin D, µg	5.6 (3.3)	9.1 (3.6)	0.14^*^	0.21^**^
α-tocopherol, mg	6.0 (1.5)	7.7 (2.4)	0.28^**^	0.39^**^
Dietary fiber, g	11.6 (2.6)	14.9 (4.8)	0.27^**^	0.47^**^
Salt, g	7.8 (1.4)	10.5 (3.2)	0.29^**^	0.31^**^
Cereals/potatoes/starches, g	500 (97)	452 (123)	0.23^**^	0.29^**^
Soy products, g	50.1 (30.1)	78.5 (39.1)	0.25^**^	0.28^**^
Fishes and shellfishes, g	49.8 (28.4)	65.7 (28.0)	0.20^**^	0.32^**^
Meats, g	74.8 (32.1)	97.1 (45.1)	0.30^**^	0.27^**^
Eggs, g	37.5 (21.6)	42.9 (21.7)	0.39^**^	0.33^**^
Milk and dairy products, g	278 (120)	365 (187)	0.51^**^	0.51^**^
Green and yellow vegetables, g	44.9 (25.0)	73.3 (32.2)	0.32^**^	0.40^**^
Other vegetables, g	169 (72)	237 (96)	0.36^**^	0.52^**^
Algae, g	4.3 (6.8)	5.4 (5.0)	0.42^**^	0.34^**^
Fruits, g	58.6 (79.8)	115 (104)	0.40^**^	0.46^**^
Confectioneries, g	61.5 (42.5)	83.1 (58.5)	0.31^**^	0.40^**^

FFQ, food frequency questionnaire; SD, standard deviation.

^a^Retinol equivalents.

^b^β carotene equivalents.

^c^Energy-adjusted.

^*^P < 0.05.

^**^P < 0.01.

## DISCUSSION

This cohort study will reveal determinants of cardiometabolic risk factors and cancer risk factors during childhood. The study will also provide data on the tracking of environmental and lifestyle behaviors and their association with some health conditions included as secondary endpoints. We assessed the validity of a FFQ modified for use in follow-up surveys and obtained results similar to those of the FFQ for 6-year-old children.^25^

The longitudinal design and high response rate at the baseline are strengths of the study. A high follow-up rate would be expected because it is a school-based study. Other strengths include the collection of dietary data using a validated tool; the inclusion of biochemical variables; and the measurement of a large number of predictor variables, confounding factors, and proxy outcomes for later diseases. In particular, obtaining fasting blood samples is advantageous.

Several limitations should be considered. Our FFQ had generally weak-to-moderate correlations with diet records. However, the results of our study were similar to those of previous studies,^20^^,^^21^^,^^32^^–^^36^ which indicates that assessing the diets of children using a FFQ is difficult. For example, the correlation coefficients for total fat ranged from 0.15^36^ to 0.62^32^ in previous studies. High correlations (*r* > 0.5) were obtained in studies assessing the diet for a short period (4 weeks).^20^^,^^32^ Nonetheless, we should admit that our FFQ is not valid for estimating some nutrients, such as cryptoxanthin and vitamin B1. At the baseline, we were unable to collect blood samples to measure cardiometabolic biomarkers, which is a major limitation of the study. Thus, a prospective design will be applied for the changes of biomarkers between the second and third follow-up surveys. Our sample size is small for rare events, such as hyperlipidemia and hypertension. Therefore, in the use of biomarkers, broader categories including borderline levels^37^ should be considered. Our sample size would be sufficient for common events. For example, to assess the association between diet and the onset of menarche, the sample size of 1,000 girls provides sufficient power (>80%) to detect a hazard risk >1.5 (or <0.67) for the highest versus lowest tertiles of dietary intakes, assuming that 58% of them reach menarche before the survey during the first grade of junior high school.^38^ The source population was children in Hekinan City, and we obtained a relatively high participation rate, although a high response rate is not necessarily linked to a reduction in the nonresponse bias.^39^ Nonetheless, our study population is not representative of the population of children in Japan, which affects the generalizability of our findings. Data collection regarding biomarkers, as well as detailed lifestyle and environment information, is planned only twice during the follow-up, which might cause misclassification bias. We cannot confirm self-reported information, such as medical history, allergy symptoms, and frequency of visit to clinics, with data from medical records.

In conclusion, the present study provides a unique opportunity to evaluate the associations between early life exposures and major risk factors of chronic diseases. The study has the potential to provide new insight into the etiology of chronic diseases and to assist in planning primary prevention strategies for these diseases in the early stage of life.

## APPENDIX A. SUPPLEMENTARY DATA

eTable 1. Characteristics of 1639 boys at baseline

eTable 2. Characteristics of 1,428 girls at baseline

eTable 3. Daily nutrient and food intakes among 1,623 boys

eTable 4. Daily nutrient and food intakes among 1,408 girls
