# Supplementary material for: The Hekinan Children’s Study: Design and Profile of Participants at Baseline
Source: J Epidemiol. 2019 Jul 5;29(7):272–7. doi: 10.2188/jea.JE20180005 (PMC6556436; doi:10.2188/jea.JE20180005)
Supplement: Supplementary file 1 [file je-29-272-s001.pdf]

eTable 1. Characteristics of 1639 boys at baseline

	Number of v response	Mean (SD)	Number (%)
Year	1,639		
2011			367 (22.4)
2012			295 (18.0)
2013			327 (20.0)
2014			327 (20.0)
2015			323 (19.7)
Age, years	1,639	6.99 (0.28)	
Height, cm	1,625	118.3 (4.9)	
Weight, kg	1,625	21.5 (3.6)	
BMI, kg/m^2	1,625	15.3 (1.8)	
Height at birth, cm	1,579	49.3 (2.6)	
Weight at birth, g	1,596	3019 (449)	
Height at 4 months, cm	1,534	63.0 (3.0))	
Weight at 4 months, kg	1,535	6.9 (0.9)	
Height at 1.5 years, cm	1,501	80.3 (3.0)	
Weight at 1.5 years, kg	1,507	10.6 (1.1)	
Height at 3 years, cm	1,476	93.3 (3.4)	
Weight at 3 years, kg	1,477	13.7 (1.6)	
Number of sibling	1,632	1.4 (0.9)	
Breast feeding[a]	1,612		1557 (96.6)
Users of cellar phone	1,637		96 (5.9)
Users of dietray supplements	1,612		137 (8.5)
Nonusers of sunscreen	1,633		1102 (67.5)
Medical history			
Heart or kidney disease	1,636		59 (3.6)
Digestive diseases	1,635		16 (1.0)
Asthma	1,567		220 (14.0)
Hospitalization in the past year	1,627		70 (4.3)
Number of visits to clinics in the past year	1,496	7.5 (8.8)	
Number of fever episodes in the past year	1,615	2.5 (2.2)	
Number of common cold events/influenza infe(in the past year	1,611	3.7 (3.7)	
Diet			
Breakfast			
Not every day	1,635		119 (7.3)
Eat alone	1,630		94 (5.8)

Timing at weekdays	1,613	6:50 a.m. (0:17)	
Timing at weekends	1,586	7:50 a.m. (0:43)	
Dinner			
Not every day	1,637		4 (0.2)
Eat alone	1,632		1(0.06)
Timing at weekdays	1,615	6:35 p.m. (0:38)	
Timing at weekends	1,595	6:35 p.m. (0:36)	
Fast foods, ≥once a week	1,637		97 (5.9)
Sleep			
Wake-up time at weekdays	1,636	6:32 a.m. (0:20)	
Wake-up time at weekends	1,631	7:13 a.m. (0:45)	
Bed-time at weekdays	1,636	9:12 p.m. (0:36)	
Bed-time at weekends	1,636	9:29 p.m. (0:41)	
Duration of sleep at weekdays, hours	1,635	9.3 (0.6)	
Duration of sleep at weekends, hours	1,630	9.7 (0.7)	
Physical activity			
Moderate-vigorous, h/wk	1,630	2.26 (3.03)	
Playtime score	1,611	11.0 (6.1)	
Strength and Difficulties Questionnaire score	1,636	11.2 (5.3)	
Fathers			
Age, years	1,539	38.5 (5.4)	
Height, cm	1,527	170.8 (5.7)	
BMI, kg/m^2	1,446	23.4 (3.3)	
Years of education ≥16 years	1,511		414 (27.4)
Current smokers	1,547		711 (46.0)
Ex-smokers	1,547		372 (24.1)
Mothers			
Age, years	1,615	36.4 (4.8)	
Height, cm	1,607	157.5 (5.4)	
Weight, kg	1,526	52.4 (8.0)	
BMI, kg/m^2	1,524	21.2 (3.0)	
Years of education ≥16 years	1,587		265 (16.7)
Current smokers	1,588		220 (13.9)
Ex-smokers	1,588		229 (14.4)

BMI, body mass index; SD, standard deviation.

[a]Including mixed feeding

eTable 2. Characteristics of 1,428 girls at baseline

	Number of v response	Mean (SD)	Number (%)
Year	1,428		
2011			299 (20.9)
2012			286 (20.0)
2013			318 (22.3)
2014			279 (19.5)
2015			246 (17.2)
Age, years	1,428	6.99 (0.28)	
Height, cm	1,424	117.6 (4.9)	
Weight, kg	1,424	21.2 (3.4)	
BMI, kg/m^2	1,424	15.2 (1.7)	
Height at birth, cm	1,372	48.8 (2.3)	
Weight at birth, g	1,391	2939 (430)	
Height at 4 months, cm	1,324	61.5 (2.6)	
Weight at 4 months, kg	1,326	6.4 (0.8)	
Height at 1.5 years, cm	1,305	79.0 (3.0)	
Weight at 1.5 years, kg	1,310	10.0 (1.1)	
Height at 3 years, cm	1,283	92.2 (3.4)	
Weight at 3 years, kg	1,287	13.3 (1.5)	
Number of sibling	1,424	1.4 (0.8)	
Breast feeding[a]	1,401		1336 (95.4)
Users of cellar phone	1,424		126 (8.9)
Users of dietray supplements	1,404		113 (8.1)
Nonusers of sunscreen	1,419		699 (49.3)
Medical history			
Heart or kidney disease	1,422		58 (4.1)
Digestive diseases	1,421		4 (0.3)
Asthma	1,382		130 (9.4)
Hospitalization in the past year	1,418		58 (4.1)
Number of visits to clinics in the past year	1,302	7.3 (10.1)	
Number of fever episodes in the past year	1,407	2.3 (2.2)	
Number of common cold events/influenza infe(in the past year	1,400	3.7 (4.1)	
Diet			
Breakfast			
Not every day	1,418		83 (5.9)
Eat alone	1,412		69 (4.9)

Timing at weekdays	1,404	6:47 a.m. (0:17)	
Timing at weekends	1,377	7:51 a.m. (0:41)	
Dinner			
Not every day	1,417		6 (0.4)
Eat alone	1,414		1 (0.07)
Timing at weekdays	1,401	6:34 p.m. (0:36)	
Timing at weekends	1,381	6:35 p.m. (0:35)	
Fast foods, ≥once a week	1,421		73 (5.1)
Sleep			
Wake-up time at weekdays	1,423	6:30 a.m. (0:20)	
Wake-up time at weekends	1,422	7:23 a.m. (0:44)	
Bed-time at weekdays	1,425	9:10 p.m. (0:35)	
Bed-time at weekends	1,421	9:27 p.m. (0:39)	
Duration of sleep at weekdays, hours	1,422	9.3 (0.5)	
Duration of sleep at weekends, hours	1,417	9.9 (0.7)	
Physical activity			
Moderate-vigorous, h/wk	1,417	0.95 (1.48)	
Playtime score	1,394	10.8 (6.0)	
Strength and Difficulties Questionnaire score	1,423	10.0 (5.0)	
Fathers			
Age, years	1,343	38.5 (5.5)	
Height, cm	1,323	170.8 (5.9)	
BMI, kg/m^2	1,254	23.4 (3.3)	
Years of education ≥16 years	1,313		358 (27.3)
Current smokers	1,350		613 (45.4)
Ex-smokers	1,350		350 (25.9)
Mothers			
Age, years	1,407	36.4 (4.6)	
Height, cm	1,399	157.5 (5.2)	
Weight, kg	1,329	52.3 (8.3)	
BMI, kg/m^2	1,329	21.1 (3.1)	
Years of education ≥16 years	1,389		238 (17.1)
Current smokers	1,391		162 (11.7)
Ex-smokers	1,391		200 (14.4)

BMI, body mass index; SD, standard deviation.

[a]Including mixed feeding

eTable 3. Daily nutrient and food intakes among 1,623 boys

	Mean (SD)
Energy, kcal	1,687 (442)
Protein, g	63.0 (17.8)
Total fat, g	56.5 (17.3)
Saturated fat, g	20.1 (6.3)
Monounsatulated fat, g	19.2 (6.2)
Polyunsatulated fat, g	10.2 (3.4)
Cholesterol, mg	287 (101)
Carbohydrate, g	229 (60)
Calcium, mg	714 (310)
Magnesium, mg	225 (70)
Phosphorus, mg	1,078 (324)
Iron, mg	6.4 (2.1)
Zinc, mg	7.8 (2.1)
Sodium, mg	3,108 (1033)
Potassium, mg	2,442 (783)
Vitamin A,[a] μg	649 (268)
Retinol, μg	320 (200)
α-carotene, μg	744 (333)
β-carotene, μg	3,363 (1430)
Cryptoxantin, μg	337 (465)
Carotene,[b] μg	3,916 (1642)
Vitamin B1, mg	0.93 (0.27)
Vitamin B2, mg	1.35 (0.46)
Vitamin B6, mg	1.11 (0.35)
Vitamin B12, μg	5.70 (2.57)
Folate, μg	280 (100)
Niacin, mg	13.0 (4.0)
Vitamin C, mg	92 (42)
Vitamin D, μg	6.4 (2.8)
α-tocopherol, mg	6.2 (2.19
Dietary fiber, g	11.3 (3.9)
Salt, g	7.9 (2.6)
Cereals/potatos/starches, g	308 (90)
Soy products, g	55.3 (46.0)
Fishes and shellfishes, g	45.1 (21.8)
Meats, g	66.9 (27.8)

Eggs, g	29.2 (15.7)
Milk and dairy products, g	360 (204)
Green and yellow vegetables, g	59.5 (29.5)
Other vegetables, g	186 (89)
Algae, g	3.6 (3.2)
Fruits, g	99 (99)
Confectioneries, g	73 (58)

SD, standard deviation.

[a] Retinol equivalents

[b] β carotene equivalents

eTable 4. Daily nutrient and food intakes among 1,408 girls

	Mean (SD)
Energy, kcal	1,571 (419)
Protein, g	59.2 (16.6)
Total fat, g	52.8 (16.1)
Saturated fat, g	18.5 (5.7)
Monounsatulated fat, g	17.9 (5.8)
Polyunsatulated fat, g	9.7 (3.2)
Cholesterol, mg	272 (96)
Carbohydrate, g	216 (66)
Calcium, mg	661 (247)
Magnesium, mg	216 (66)
Phosphorus, mg	1,010 (290)
Iron, mg	6.3 (2.1)
Zinc, mg	7.4 (2.0)
Sodium, mg	2,938 (981)
Potassium, mg	2,343 (728)
Vitamin A,[a] μg	635 (244)
Retinol, μg	303 (175)
α-carotene, μg	734 (311)
β-carotene, μg	3,396 (1390)
Cryptoxantin, μg	367 (507)
Carotene,[b] μg	3,396 (1390)
Vitamin B1, mg	0.88 (0.26)
Vitamin B2, mg	1.26 (0.40)
Vitamin B6, mg	1.07 (0.33)
Vitamin B12, μg	5.42 (2.42)
Folate, μg	279 (100)
Niacin, mg	12.5 (3.9)
Vitamin C, mg	94 (44)
Vitamin D, μg	6.1 (2.6)
α-tocopherol, mg	6.0 (2.1)
Dietary fiber, g	11.2 (3.9)
Salt, g	7.5 (2.5)
Cereals/potatos/starches, g	281 (85)
Soy products, g	55.2 (38.5)
Fishes and shellfishes, g	43.7 (20.2)
Meats, g	61.5 (26.0)

Eggs, g	28.1 (14.7)
Milk and dairy products, g	320 (156)
Green and yellow vegetables, g	63.3 (32.0)
Other vegetables, g	187 (83)
Algae, g	4.0 (3.4)
Fruits, g	99 (96)
Confectioneries, g	70 (52)

SD, standard deviation.

[a] Retinol equivalents

[b] β carotene equivalents
